# The impact of stress on tumor growth: peripheral CRF mediates tumor-promoting effects of stress

**DOI:** 10.1186/1476-4598-9-261

**Published:** 2010-09-27

**Authors:** Alicia Arranz, Maria Venihaki, Berber Mol, Ariadne Androulidaki, Erini Dermitzaki, Olga Rassouli, Jorge Ripoll, Efstathios N Stathopoulos, Rosa P Gomariz, Andrew N Margioris, Christos Tsatsanis

**Affiliations:** 1 Department of Clinical Chemistry, School Of Medicine, University of Crete, 71003 Heraklion, Greece; 2 Department of Pathology, School Of Medicine, University of Crete, 71003 Heraklion, Greece; 3 Institute for Electronic Structure and Laser, Foundation for Research and Technology-Hellas, 71110 Heraklion, Greece; 4 Department of Cell Biology, Faculty of Biology, Complutense University, Madrid, Spain

**Keywords:** Corticotropin Releasing Hormone, stress, 4T1, breast cancer

## Abstract

**Introduction:**

Stress has been shown to be a tumor promoting factor. Both clinical and laboratory studies have shown that chronic stress is associated with tumor growth in several types of cancer. Corticotropin Releasing Factor (CRF) is the major hypothalamic mediator of stress, but is also expressed in peripheral tissues. Earlier studies have shown that peripheral CRF affects breast cancer cell proliferation and motility. The aim of the present study was to assess the significance of peripheral CRF on tumor growth as a mediator of the response to stress in vivo.

**Methods:**

For this purpose we used the 4T1 breast cancer cell line in cell culture and in vivo. Cells were treated with CRF in culture and gene specific arrays were performed to identify genes directly affected by CRF and involved in breast cancer cell growth. To assess the impact of peripheral CRF as a stress mediator in tumor growth, Balb/c mice were orthotopically injected with 4T1 cells in the mammary fat pad to induce breast tumors. Mice were subjected to repetitive immobilization stress as a model of chronic stress. To inhibit the action of CRF, the CRF antagonist antalarmin was injected intraperitoneally. Breast tissue samples were histologically analyzed and assessed for neoangiogenesis.

**Results:**

Array analysis revealed among other genes that CRF induced the expression of SMAD2 and β-catenin, genes involved in breast cancer cell proliferation and cytoskeletal changes associated with metastasis. Cell transfection and luciferase assays confirmed the role of CRF in WNT- β-catenin signaling. CRF induced 4T1 cell proliferation and augmented the TGF-β action on proliferation confirming its impact on TGFβ/SMAD2 signaling. In addition, CRF promoted actin reorganization and cell migration, suggesting a direct tumor-promoting action. Chronic stress augmented tumor growth in 4T1 breast tumor bearing mice and peripheral administration of the CRF antagonist antalarmin suppressed this effect. Moreover, antalarmin suppressed neoangiogenesis in 4T1 tumors in vivo.

**Conclusion:**

This is the first report demonstrating that peripheral CRF, at least in part, mediates the tumor-promoting effects of stress and implicates CRF in SMAD2 and β-catenin expression.

## Background

Stress has been described as a promoter of tumor growth and angiogenesis in different *in vivo *models [1]. Thus, it has been considered that during chronic stress and depression, the persistent activation of the hypothalamic-pituitary-adrenal (HPA) axis is probably responsible of an impaired immune response, contributing to the development and progression of several types of cancer [2].

Corticotropin Releasing Factor (CRF) was the first peptide isolated from the now named CRF-related peptides family that also includes urocortin 1, urocortin 2 and urocortin 3. This family of peptides exerts its biological actions through the activation of two receptors: CRF receptor 1 (CRF_1_) and CRF receptor 2 (CRF_2_). CRF exert its effect primarily via CRF receptor 1 and at a lesser extent via CRF_2 _[3], exhibiting a 10 fold higher affinity for the former.

CRF has been described to be present not only in the central nervous system, its primary site of expression, but also in peripheral tissues and organs [3]. Indeed, multiple studies have shown that CRF mediates endocrine responses to stress, not only by activating the HPA axis but also via direct actions in the periphery [4-6]. In this regard, the CRF-based paracrine activity has been postulated to participate in the modulation of stress effects on the gastrointestinal system [5]. Moreover, CRF-related peptides exert direct actions on cardiomyocytes mediating the adaptive response of the cardiovascular system to stressful conditions such as ischemia and reperfusion [7,8].

In the tumor microenvironment, CRF is released by endothelial and immune cells and by the local neuronal innervation [9-11]. Moreover, peptides of the CRF family and their receptors have been also found expressed by several cancer cells [12], such as human renal cell carcinoma [13], tumorous adrenocortical cells [14], human endometrial, prostate, ovarian and breast cancer cells [14-19], human pheochromocytoma cells and melanomas [20-22] and the murine melanoma cell line B16F10 [23]. However, the effects exerted by CRF in cancer cells range from promotion of cancer cell proliferation and migration to inhibition of proliferation and induction of angiogenesis. Thus, CRF has been described to inhibit cell proliferation via CRF1 in the endometrial adenocarcinoma cell line Ishikawa [24] and in the human HaCaT keratinocytes [25]. In contrast, in the Y79 retinoblastoma cell line CRF suppresses apoptosis via downregulation of pro-caspase 3 cleavage and activation [26] and in the B16F10 murine melanoma cell line it enhances cell migration through the ERK1/2 pathway [23]. Moreover, in the human breast cancer MCF7 cells, an estrogen-dependent tumor cell line, CRF inhibits cell proliferation but promotes motility and invasiveness via the activation of CRF_1 _[17,18]. In addition, CRF induces local immunosuppression by promoting apoptosis of cytotoxic T-cell via the prduction of Fas ligand (FasL) in ovarian cancer cells [19].

The aim of the present study was to test the role of peripheral CRF as a mediator of stress response on breast cancer cell growth using both *in vivo *and *in vitro *studies on the 4T1 breast cancer cell line. In the first part of this work we evaluated the direct effects of CRF on this cell line in culture. In the second part, we used a mouse model of orthotropic injection of breast cancer cells in the mammary fat pad of Balb/c mice. In this model we studied the effect of stress on tumor growth and we evaluated the impact of inhibition of peripheral CRF. For this purpose we administered antalarmin intraperitoneally, which does not affect stress-induced Hypothalamus-pituitary-adrenal (HPA) axis responses [27]. In this way, we determined the effect of peripheral CRF inhibition on tumor growth in the presence or absence of stress exposure.

Our results showed that CRF increased proliferation, migration and actin polymerization in 4T1 cells. Moreover, it modified the expression of several molecules involved in tumor growth and metastasis. Two of them, SMAD2 and β-Catenin, transcription factors connected with the TGFβ and the Wnt signaling pathways respectively [28,29], were increased following CRF treatment. Finally, *in vivo *studies demonstrated that peripheral CRF induced angiogenesis and tumor growth *in vivo*.

## Results

### 1. Expression of CRF receptors in 4T1 cells

**Figure 1:**
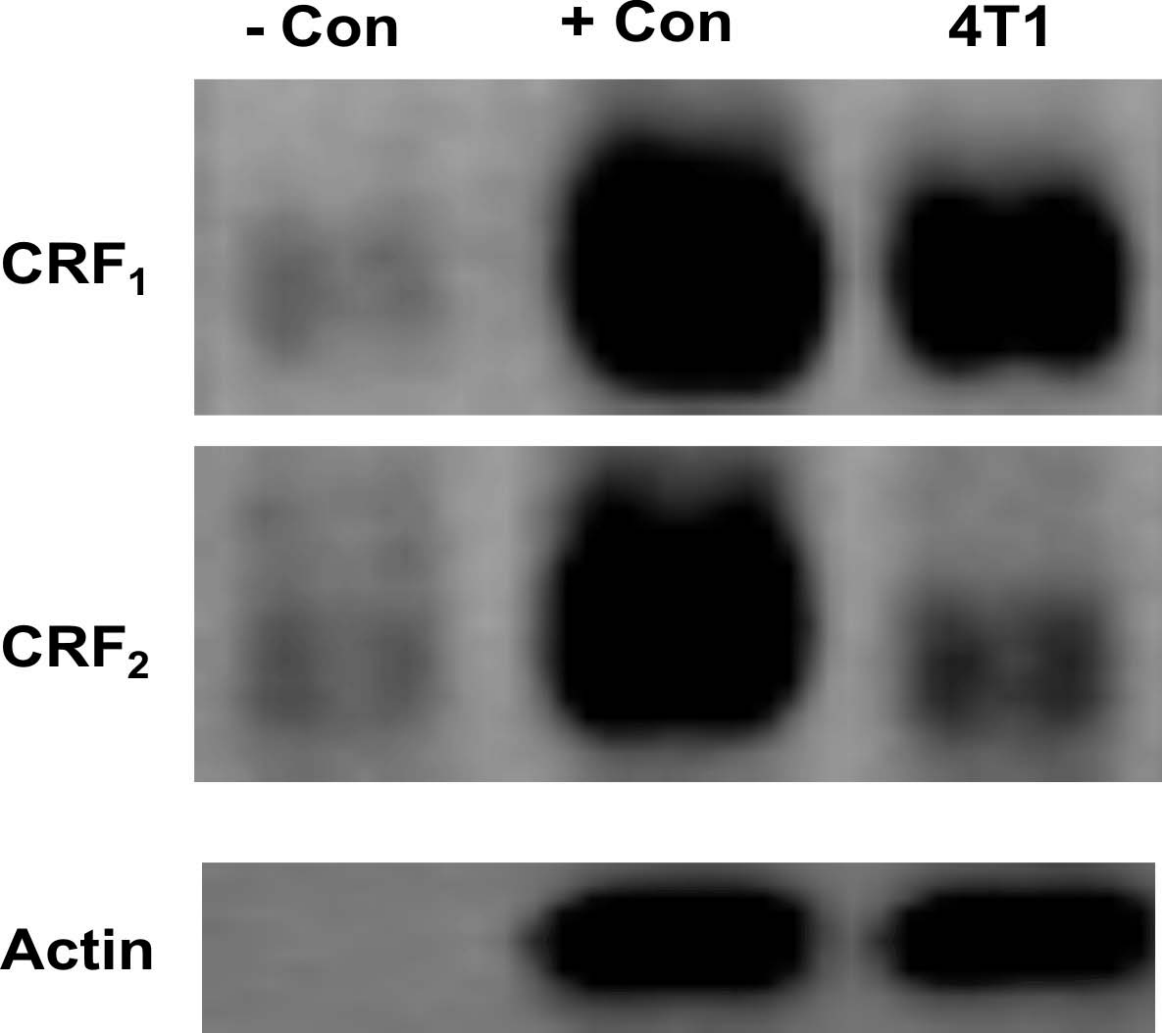
**Expression of CRF receptors in 4T1 cells**. CRF receptor 1 and low levels of CRF receptor 2 were found expressed in 4T1 cells.- Con, negative control (non rt); + Con, positive control (mouse brain).

The expression of CRF receptors in 4T1 cells has not been previously reported. To asses any possible direct effect of CRF in 4T1 cells, our first aim was to investigate the expression of CRF receptor 1 and 2 in this cell line. Our results confirmed that 4T1 cells expressed high levels of CRF_1 _receptor and very low levels of CRF_2 _receptor type b (CRF_2b_) (**Figure **1). Similarly, previous studies from our group had shown that MCF7 breast cancer cells also express CRF_1 _receptor and low levels of CRF_2 _[18].

### 2. CRF induces proliferation of 4T1 cells in a time-dependent manner

**Figure 2:**
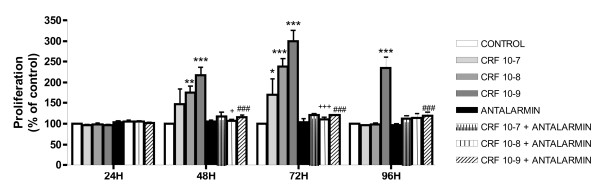
**Effects of CRF on 4T1 cells proliferation**. 4T1 cells were treated with different doses of CRF for 48 to 96 hours. CRF significantly induced proliferation of 4T1 cells, as measured by the MTT assay. The CRF_1 _antagonist Antalarmin reversed this effect. Data are expressed as the MEAN ± SEM of three independent experiments. * p < 0.05 compared to untreated control cells, + p < 0.05 compared to cells treated with 10^-8^M CRF and # p < 0.05 compared to cells treated with 10^-9^M CRF.

Regulation of cancer cell proliferation is readily associated with malignancy. CRF has been previously described to reduce proliferation of cancer cell lines such as Ishikawa endometrial carcinoma cells, pheochromocytoma cell lines and the breast cancer cell line MCF7 [17,18,20,21,24]. In the Y79 retinoblastoma cell line, however, CRF suppresses apoptosis [26]. To asses the effect of CRF on 4T1 cell proliferation, 4T1 cells were treated with different doses of CRF for different time points. The results indicated that CRF promoted 4T1 cell proliferation with the most effective dose being 10^-9 ^M being evident at 48, 72 and 96 hours (**Figure **2). No effect on proliferation was observed at 24 hours. To determine if this effect was abrogated by the CRF1 antagonist Antalarmin, we treated cells with different concentrations of CRF for in the presence or absence of Antalarmin for the same time periods. The results indicated that CRF promoted 4T1 proliferation via CRF1 receptor (**Figure **2).

### 3. CRF affects the expression of molecules involved in tumor cell growth and metastasis: induction of β-catenin and SMAD2 in a time-dependent manner

To further evaluate the effect of CRF in tumor cell growth and metastasis in our system, RNA from 4T1 cells untreated and treated with 10^-8^M CRF at the indicated time points was analyzed using a gene-specific oligo microarray for 113 genes known to be involved in tumor growth and metastasis (Superarray, Qiagen). Image data were transformed into numerical and into color intensity data as described in Materials and methods.

**Figure 3:**
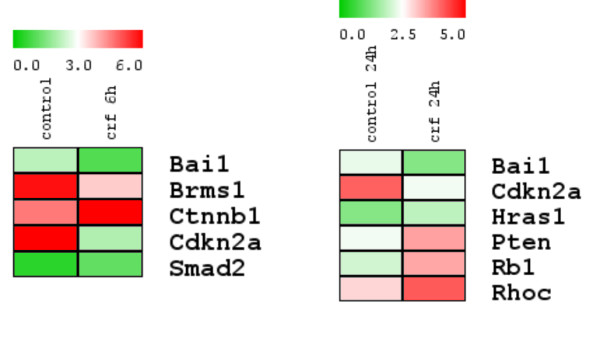
**Effects of CRF on the expression of molecules involved in tumor growth and metastasis**. Gene-specific oligo microarrays for 113 genes known to be involved in tumor growth and metastasis were performed as described in Materials and methods. Effect of CRF on the expression of these molecules in 4T1 cells was analyzed. Mean expression of each gene was transformed into color intensity using the program TIGR MultiExperiment Viewer V4.5.1. The minimal value (green) and the maximum (red) were considered the limits (as noticed in bar above). The figure shows the molecules which expression was affected by CRF after 6 or 24 h of treatment: Bai1, Brain-specific angiogenesis inhibitor 1; Brms1, Breast cancer metastasis-suppressor 1; Ctnnb1, β-Catenin; Cdkn2a, Cyclin-dependent kinase inhibitor 2A; Smad2, MAD homolog 2; Hras1, Harvey rat sarcoma virus oncogene 1; Pten, Phosphatase and tensin homolog; Rb1, Retinoblastoma 1; Rhoc, Ras homolog gene family, member C.

The ratio of gene expression in CRF-treated to untreated cells was used to determine increased or decreased RNA expression of genes after CRF treatment. Our data showed that CRF modifies the expression of several molecules involved in tumor cell growth and metastasis that can be classified in groups according to function as shown in **Table **1. **Figure **3 illustrates the color intensity analysis according to the expression levels of genes affected by CRF treatment.

**Table 1:** List of genes affected by CRF

Functional groups	GenBank	Name	Symbol	Ratio control/CRF 6 h	Ratio control/CRF 24 h
**1, 3, 5**	NM 007614	**β-Catenin**	**Catnb**	1.43*	**1.00**
**2, 3**	NM 008284	**Harvey rat sarcoma virus oncogene 1**	**Hras1**	**1.00**	1.41*
**2, 4**	NM 008960	**Phosphatase and tensin homolog**	**Pten**	**1.00**	1.44*
**2, 5**	NM 009029	**Retinoblastoma 1**	**Rb1**	**0.97**	1.65*
**3**	NM 007484	**Ras homolog gene family, member C**	**Rhoc**	**1.01**	1.42*
**5**	NM 010754	**MAD homolog 2**	**Smad2**	2.32*	**0.98**
**6**	NM 174991	**Brain-specific angiogenesis inhibitor 1**	**Bai1**	0.44*	0.57*
**2, 3**	NM 134155	**Breast cancer metastasis-suppressor 1**	**Brms1**	0.60*	**0.85**
**2, 5**	NM 009870	**Cyclin-dependent kinase inhibitor 2A**	**Cdkn2a**	0.31*	0.59*

Functional groups: 1, Cell adhesion; 2, Cell cycle; 3, Cell growth and proliferation; 4, apoptosis; 5, Transcription factors and regulators; 6, Other genes involved in metastasis. *, increase or decrease equal or higher than 40%

Interestingly, our results with the oligo-microarrays pointed out the CRF-induced expression of two essential transcription factors involved in metastasis: β-catenin and SMAD2. To confirm these results, western blot were performed as described in Materials and methods.

**Figure 4:**
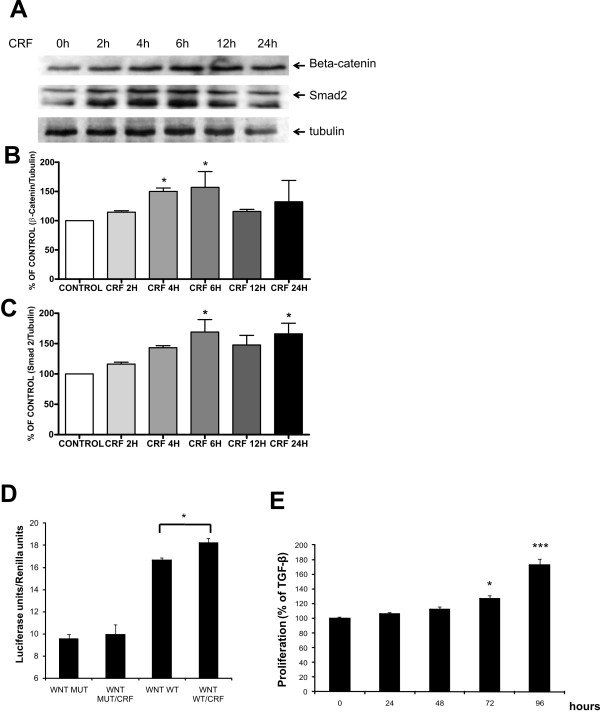
**CRF induced the expression of β-catenin and SMAD2**. Western blot analysis were performed as described in Materials and methods in order to asses the effect of CRF on β-catenin and SMAD2 expression at protein levels. CRF significantly induced the expression of β-catenin (A,B) at 4 and 6 h whereas the expression of SMAD2 (A, C) was significantly induced at 6, and 24 h. Data are expressed as MEAN ± SEM of five independent experiments. * p < 0.05 compared to untreated control. D. 4T1 cells were transfected with a luciferase construct containing a WNT-responsive element in which the complex of β-catenin and Tcf binds. Cells were stimulated with CRF and luciferase activity was measured in cell lysates 24 hours following stimulation. Data are expressed as MEAN ± SEM of three independent experiments (*p < 0.05). E. 4T1 cells were stimulated with TGFβ at 5 ng/ml in the presence or absence of CRF at 10^-8^M and proliferation was measured using the MTT assay. Data are expressed as MEAN ± SEM of three independent experiments. * p < 0.05 compared to TGFβ -only treated cells.

The potential effect of CRF on β-catenin and subsequently Wnt signaling may confer a novel mechanism for crosstalk between breast cancer cells and stress neuropeptides. Our results with western blot confirmed that CRF rapidly induced β-catenin expression at the protein level (**Figure **4A, B). Up-regulation of SMAD2, a downstream mediator of TGF-β signaling [28] was also confirmed by western blot analysis (**Figure **4B, C).

To address the functional significance of the induction of β-catenin in 4T1 cells, we transfected 4T1 cells with a WNT reporter construct containing Tcf binding elements upstream the luciferase gene and treated them with CRF. The results indicated that CRF treatment augmented WNT signaling, confirming the functional significance of β-catenin induction. The effect was abrogated when the Tcf binding consensus was mutated (Figure 4D). To confirm the importance of CRF-induced Smad2 expression, we assessed the effect of CRF on TGFβ signaling. 4T1 cells were treated with TGFβ in the presence or absence of CRF and cell proliferation was measured. The results indicated that CRF augmented TGFβ-induced proliferation of 4T1 cells (Figure 4E).

### 4. CRF increased actin polymerization in 4T1 cells

It has been reported that TGF-β and β-catenin are involved in cell motility and invasiveness in epithelial cancer cells and in cytoskeletal changes, respectively [30]. Since our results showed that the expression of β-catenin and SMAD2 is increased in 4T1 cells by CRF, we therefore examined the impact of CRF on cytoskeletal changes in this cell line.

**Figure 5:**
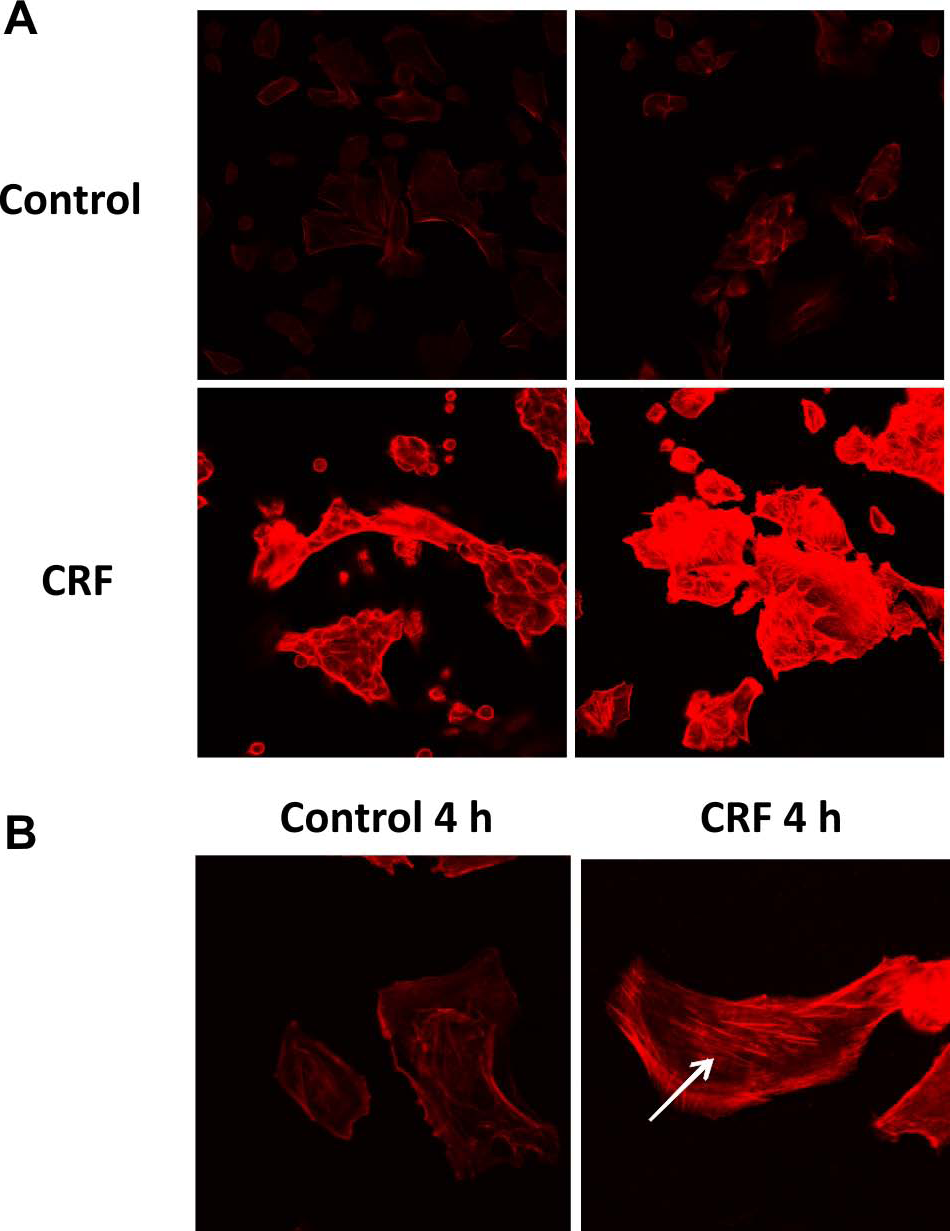
**Effect of CRF on actin polymerization in 4T1 cells**. 4T1 cells were cultured on coverglass and stimulated with CRF for 2 or 4 h. After fixation, cells were permeabilized and stained for F-actin with rhodamine-conjugated phalloidin, as described in Materials and methods. CRF treatment resulted in a more intense actin staining compared to untreated control, especially after 4 h (A). Moreover, CRF treated 4T1 cells showed more pronounced actin stress fibers compared to untreated control (indicated by the arrow). Pictures are representatives of two independent experiments.

**Figure 6:**
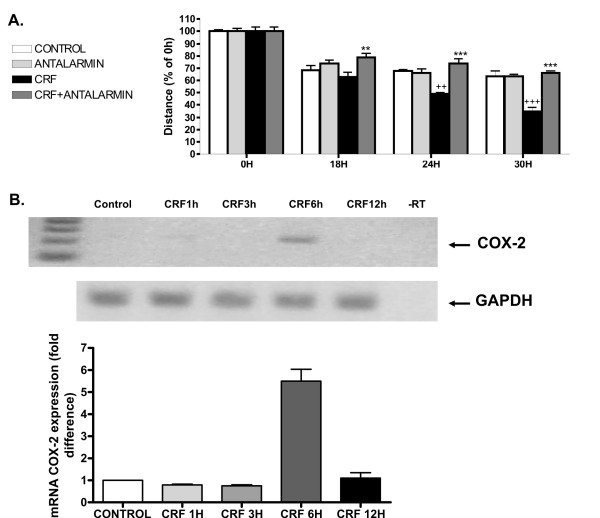
**CRF promotes migration of 4T1 cells and expression of Cox-2**. A. 4T1 cells were stimulated with 10^-8^M CRF or vehicle (control) in the presence or absence of Antalarmin and photographed at 0, 6, 12 and 24 hours after disruption of a small area of the cell layer. The remigration of the cells was quantified by measuring the distance at 3 at least different points of the gap of each image with the program Image J, and expressing the distance % of the average of the distance initially formed by the disruption (time 0). Results represent the average of three independent experiments. ++p < 0,01, +++p < 0.001 compared to control cells at the same time point; **p < 0,01; ***p < 0,001 compared to CRF-treated cells at the same time point. B. Expression of Cox-2 was measured in 4T1 stimulated cells by RT-PCR (upper panel) and real-time RT-PCR. Results represent the average of 3 independent experiments; ***p < 0,001 compared to control cells.

To this aim, 4T1 cells were treated with 2 × 10^-8^M CRF and stained with rhodamine-phalloidin, as described in Materials and methods. The toxin phalloidin, conjugated to the fluorescent dye rhodamine, binds specifically to polymerized actin allowing us to visualize the architecture of actin in the cell. Cells treated with CRF showed more intense staining compared to the untreated controls, most extensively seen after 4 h treatment (**Figure **5A). In addition, CRF treated cells showed increased actin stress fibers (**Figure **5B). The altered actin structures seen after CRF treatment might be associated with an increase in cancer cell motility, a process necessary for tumor cells to invade and metastasize. To assess the impact of CRF on 4T1 motility and migration we performed the wound healing assay, in which a gap is formed in a cell monolayer and the speed of cell migration was estimated by measuring the closure of the gap. The results indicated that CRF promoted 4T1 cell motility and migration (Figure 6A) further supporting our hypothesis. Antalarmin reversed the effect implicating CRF1 receptor.

In order of tumors to grow and cancer cells to metastasize neoangiogenesis is required. Earlier studies from our group had shown that CRF induced Cox-2 expression [31], an enzyme known to promote angiogenesis via production of prostaglandins [32]. Indeed, treatment of 4T1 cells with CRF induced Cox-2 expression suggesting a potential impact on metastasis (Figure 6B). VEGF is a key factor that promotes angiogenesis. Treatment of 4T1 cells with CRF did not result in detectable VEGF expression (data not shown), suggesting that CRF may utilize a Cox-2 dependent, VEGF-independent mechanism to promote angiogenesis.

### 5. Evaluation of the *in vivo *model of chronic stress

In order to greater extent the molecular mediators of CRF on tumor growth and the effect of peripheral CRF, we used an *in vivo *model of restraint stress (see Materials and methods) and antalarmin, a synthetic CRF_1 _receptor antagonist [33,34].

**Figure 7:**
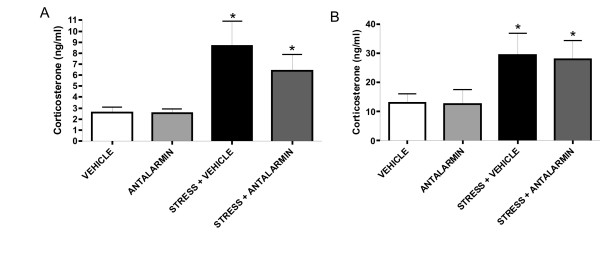
**Peripheral administration of antalarmin does not affect the HPA axis response**. As described in Materials and methods, levels of corticosterone in serum were determined by RIA in the different groups of mice immediately after the last exposure to stress and on the 4^th ^day of the interval that followed the last exposure to stress. Increased levels of corticosterone upon stress were not affected by the peripheral administration of antalarmin (A). Moreover, increased levels of corticosterone after the last exposure to stress were not affected by antalarmin either, and confirmed the development of chronic stress (B). Data are expressed as MEAN ± SEM. Five animals per group were used. * p < 0.05, compared to control not exposed to stress.

Firstly, to confirm that peripheral administration of antalarmin does not affect the role of CRF in the response of the HPA axis to stress, levels of corticosterone in serum were determined in the different groups of mice immediately after the last exposure to stress. Thus, corticosterone levels were significantly increased upon stress and were not affected by antalarmin. This suggests that when antalarmin is administered peripherally, it does not affect corticosterone production triggered by immobilization stress (**Figure **7A).

Secondly, to determine whether our experimental setup indeed resembled chronic stress, we measured corticosterone on the 4^th ^day of the interval that followed the last exposure to stress. In this manner, we confirmed that the corticosterone levels in the plasma were still increased, indicating that the mice were exposed to chonic stress. In addition, we confirmed again that antalarmin administrated intraperitoneally did not affect corticosterone production, since no difference was observed between mice injected with vehicle or antalarmin and exposed to stress (**Figure **7B).

### 6. Peripheral CRF promoted tumor growth and induced angiogenesis *in vivo*

As described in Materials and methods, six weeks after the injection of 4T1 cells into the mammary fat pad of mice, mammary glands were visualized on the animal to determine the extent of neoangiogenesis and samples were collected to perform histological analysis.

**Figure 8:**
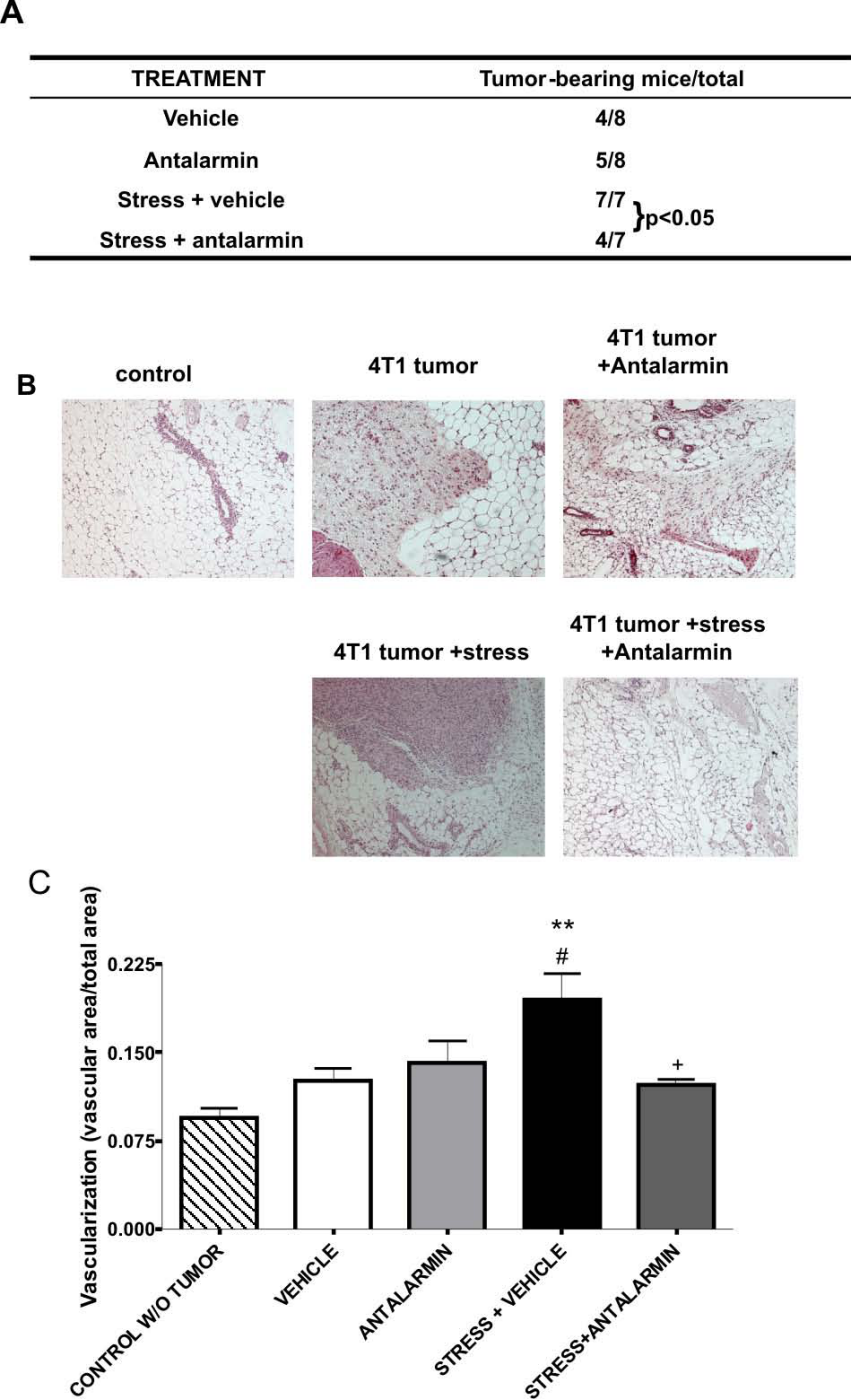
**Peripheral CRF increased tumor growth and neoangiogenesis *in vivo***. An *in vivo *model of breast tumor and restraint stress was used and the percentage of tumor bearing mice and the extend of neoangiogenesis were determined as described in Materials and methods. (A) Peripheral administration of antalarmin diminished the stress-induced increase of the percentage of tumor bearing mice. (B) Representative pictures of the histological analysis. (C) The area of vascularization is increased in animals exposed to stress but this effect is abolished by the inhibition of peripheral CRF by the ip administration of antalarmin. ** p < 0.01 compared to control without tumor. # p < 0.05 compared to tumor-bearing mice injected with vehicle. + p < 0.05 compare to tumor-bearing mice exposed to stress and injected with vehicle.

Histological and optical imaging analysis of the tumors revealed that in mice not exposed to stress, administration of antalarmin resulted in reduced tumor burden. Upon stress the percentage of tumor-bearing animals was increased compared to non-stressed animals. Administration of antalarmin in stressed animals resulted in reduction of the percentage of tumor-bearing mice **(Figure **8A). No significant difference in tumor size was observed. Histological analysis in the lung and liver revealed no metastasis in the groups analyzed (data not shown). Representative photographs of mammary tissues stained with Haematoxylin-Eosin are shown in **Figure **8B.

Angiogenesis is a hallmark of tumor growth and metastasis. Recent studies have indicated that CRF affects neoangiogenesis and that CRF_1 _mediates this effect [35]. We therefore evaluated the extent of neoangiogenesis in the 4T1 tumors and the impact of stress and CRF inhibition. To quantitatively measure angiogenesis, we used an image analysis approach based on the contrast of light autofluorescence between the mammary tissue and the blood vessels. Blood vessels absorb visible light, while mammary gland and mammary tumors are strongly autofluorescent. A user-friendly software was developed in-house and used to quantify the relative area of blood vessels in the tissue. The results showed that tumor-bearing mammary glands had increased angiogenesis compared to normal mammary glands and angiogenesis was significantly increased when mice were exposed to stress **(Figure **8C). Treatment of mice exposed to stress with antalarmin resulted in reduced angiogenesis. Our results suggest that stress augments neoangiogenesis in breast tumors and a potential mediator is peripheral CRF, since treatment with antalarmin suppressed stress-induced neoangiogenesis.

## Discussion

The impact of stress on the development of cancer has been widely proposed [1,2,36]. The stress response involves the activation of cascades in both the central and the peripheral nervous systems. CRF is the main hypothalamic stress-induced neuropeptide and its peripheral effect has also been reported in several systems [3,5,8,9,31,37-39]. Thus, the objective of this work was to analyze the role of peripheral CRF as a mediator of stress effects on cancer cells in a murine model of breast cancer. To this aim, we first analyzed the expression of CRF receptors in 4T1 cells in order to assess any direct effect of CRF on this system. In the present study we found that 4T1 cells expressed high levels of CRF_1 _receptor and low levels of CRF_2b _receptor. The expression of CRF receptors have been described in other cancer cell lines. In fact, previous studies from our group had shown that MCF7 breast cancer cells also express CRF_1 _receptor and low levels of CRF_2_. However, in the present work we found that in 4T1 cells CRF induced cell proliferation, whereas in MCF7, and others cell lines such as the adenocarcinoma cell line Ishikawa and the human HaCaT keratinocytes [18,24,25], proliferation was suppressed by CRF. In contrast, CRF induced proliferation of the At20 corticotrophic adenocarcinoma cell line and primary canine corticotrophic adenoma cells [40,41]. This discrepancy is in accordance with previous works describing that the phenotypic effects of CRF on cell proliferation were dependent on both cell type and nutrition conditions [42]. Therefore using non malignant cells it has been shown that CRF stimulated dermal fibroblasts proliferation while it inhibited cell proliferation in keratinocytes [42].

Since different reports support positive or negative actions of CRF on cancer cell growth and metastasis we assessed the effect of CRF on the expression pattern of genes involved in cancer cell metastasis. For this purpose we used gene-specific oligo microarrays. Our results demonstrated that CRF treatment increased expression of Smad2 and β-catenin, and suppressed the expression of the angiogenesis inhibitor Bai1, the metastasis suppressor Brms1 and the cell cycle regulator Cdkn2a/p16. In addition, CRF also enhanced the expression of molecules involved in cell cycle, proliferation and apoptosis, such as Ha-ras1, Myb, Pten, Rb1 and RhoC. Our studies focused on the impact of CRF on SMAD2 and β catenin, being molecules involved in two central signaling pathways regulating breast cancer growth and metastasis, these of TGFβ and Wnt respectively [28,29].

We therefore confirmed the effects of CRF on SMAD2 and β-catenin expression at protein levels. SMAD2 and β-catenin are two major transcription factors involved in metastasis. SMAD2, together with SMAD3, is associated with the TGF-β receptor. When TGF-β binds to its receptor, SMAD2 and SMAD3 are phosphorylated and form a complex with SMAD4 that translocates to the nucleus. In the nucleus, an activated SMAD complex is formed which regulates gene expression and ultimately cell growth [28]. Regarding β-catenin, apart from being a cell-cell adhesion protein, is also an important signal transduction molecule in the Wnt signaling pathway [43]. Induction of Wnt signaling, mostly by affecting β-catenin, has been described as a hallmark of colon, breast, prostate and ovarian cancer [29]. Interestingly, recent evidence described a link between the TGF-β and the Wnt-signaling pathways, since receptor-activated SMAD2 synergistically enhances the Wnt/β-catenin pathway in epithelial cancer cells [44]. Thus, the potential effect of CRF on SMAD2 and β-catenin, and subsequently TGF-β and Wnt signaling, may confer a novel mechanism for crosstalk between cancer cells and stress neuropeptides.

Moreover, it has been reported that TGF-β promotes cell motility and invasiveness in epithelial cancer cells. In addition, β-catenin is also involved in cytoskeletal changes characterized by actin polymerization, cell adhesion and motility [30]. Therefore, we analyzed the effect of CRF on actin polymerization in 4T1 cells. Our results showed higher levels of polymerized actin as well as an increase of actin stress fibers. This suggests that CRF could promote changes in cytoskeletal structures that allow cells to migrate and metastasize [45].

The results of the present and our earlier study [18] suggest distinct effects of CRF on breast cancer cells. Several reports have indicated either tumor promoting or tumor-inhibitory effects of neuropeptides. Oxytocin has been shown to suppress proliferation while ghrelin promotes proliferation in breast cancer cell lines [46,47]. Moreover, the effect of grelin on the phenotype depends on the expression of Estrogen Receptor [47,48]. In our case both MCF7 and 4T1 are ER+ cell lines suggesting that the discrepancy of the effects does not depend on ER but on other genetic differences.

Considering the results obtained *in vitro*, and our earlier studies in the human breast cancer cell line MCF7 [18], we used an *in vivo *model of breast cancer in which we exposed mice to chronic stress (see Materials and methods). Antalarmin was administered intraperitoneally and did not affect chronic stress-induced corticosterone levels ([27] and present report) but was able to inhibit its action on tumor cells. Indeed, earlier studies showed that intraperitoneal administration of antalarmin inhibited the proinflammatory role of CRF in toxin A-induced intestinal secretion and inflammation [49] or in the adjuvant induced arthritis model with Lewis rats [50]. Furthermore, inhibition of peripheral CRF with i.p. administration of antalarmin resulted in an increased survival after LPS-induced endotoxic shock, without affecting the production of corticosterone [34]. Accordingly, our results showed that administration of antalarmin intraperitoneally did not affect the elevation of corticosterone following stress exposure. Once confirmed that in our system the HPA axis was not affected, we analyzed the effects of peripheral CRF inhibition on tumor growth. We observed that i.p. administration of antalarmin in stressed animals resulted in significant reduction of tumor burden, which suggests that peripheral CRF promoted the growth or tumor cells also *in vivo*. Moreover, we quantitatively evaluated the extent of neoangiogenesis in the 4T1 tumors, as an essential process for the tumor growth and metastasis. Histological analysis did not reveal any other changes in the tumors, such as apoptotic/necrotic lesions. Our experiments showed that treatment of mice exposed to stress with antalarmin resulted in reduced angiogenesis compared to stressed mice injected with vehicle. This suggests that peripheral CRF significantly contributes to neoangiogenesis observed after stress. Moreover, our results illustrated that this effect of peripheral CRF is exerted via CRF receptor 1, since it was inhibited by the selective CRF_1 _antagonist antalarmin. Interestingly, previous reports have shown a suppressive effect of Urocortin2 on tumor vascularization via CRF receptor 2 [51,52] and depletion of CRF1 in mice suppresses intestinal angiogenesis while ablation of CRF_2 _augments it, supporting a role for CRF_1 _signals in angiogenesis [35]. Also, peripheral CRF has been shown to enhance local angiogenesis and vascular permeability in skin via a CRF receptor-dependent mechanism [10,53]. This indicates that different CRF receptors may have different effects on neoangiogenesis. Expression of Cox-2 and VEGF have been associated with neoangiogenesis. In the case of 4T1 cells CRF induced Cox-2 but not VEGF expression suggesting that it utilizes a Cox2-dependent, VEGF-independent mechanism to promote angiogenesis.

## Conclusions

Overall, this is the first report showing that CRF affects TGFβ and WNT signaling pathways, major contributors in breast tumor growth. In addition, it is the first report demonstrating *in vivo *that peripheral CRF mediates the effects of stress on breast tumor growth. Hence, this suggests that inhibition of peripheral CRF may be beneficial for suppressing stress-induced breast tumor growth.

## Experimental Procedures

### Cell Culture

The mouse mammary tumor cell line 4T1 was cultured in Dulbecco's Modified Eagle Medium (DMEM) supplemented with 10% heat-inactivated fetal bovine serum (FBS) and 1% penicillin/streptomycin (all purchased from GIBCO, UK) at 37°C in a 5% CO_2 _humidified atmosphere. For cell stimulations, 4T1 cells were plated one day before stimulation at 500.000 or 250.000 cells per well in 6-well or 24-well plates, respectively. Subsequently, medium was refreshed and supplemented with (or without for controls) synthetic rat/human CRF (Tocris, Bioscience, UK) at a concentration of 10^-8^M.

### Reverse transcriptase PCR

Total cellular RNA was isolated using Trizol reagent (Invitrogen, UK). cDNA was prepared by reverse transcriptase PCR (Superscript RT, Invitrogen) and amplified by PCR using the following primer pairs: CRFR1: fwd 5'- GCC GCC TAC AAT TAC TTC CA-3', rev 5'- CGG AGT TTG GTC ATG AGG AT - 3' and CRFR2: fwd 5'- GGA GCC CTA GTG GAG AGA CC -3', rev 5'- AGG TGG TGA TGA GGT TCC AG -3', VEGF: 5'- GTACCTCCACCATGCCAAGT-3', 5'- ACTCCAGGGCTTCATCGTTA -3', Cox-2: 5'- GCTTGCATTGATGGTGGCTG-3', 5'- CCAGATGCTATCTTTGGGGAGAC-3'. For each PCR reaction, 1 μl of cDNA was used together with primers indicated above, at 45 cycles and an annealing temperature of 60°C. 10 μl of amplified products were separated on a 1.5% agarose gel and visualized by ethidium bromide staining, or subjected to real-time PCR using SYBRgeen method as previously reported [54].

### MTT viability assay

To determine the effect of CRF on cell proliferation, MTT (3-(4,5-Dimethyl-2-thiazolyl)-2,5-diphenyl-2H-tetrazolium bromide) cell viability assays were performed. 4T1 cells were plated in flat-bottomed 96-well plates at a 5000 cells/well concentration and allowed to adhere overnight. The following day the medium was changed by fresh DMEM supplemented or not with 10^-8 ^M CRF. To determine cell growth, after 24 and 48 hours, 50 μg MTT (Sigma-Aldrich, USA) was added to each well and the plates were incubated an additional 4 h at 37°C and 5% CO_2_. After 4 h the supernatant was removed and the formed crystals were dissolved in 100 μl 0,04N HCL in isopropanol. The plates were analyzed at 570 nm with a microplate reader (Bio-Rad, UK). All assays were performed in quadruplicate and the mean values for each data point was calculated from the combined data.

### Gene expression arrays

Total RNA from 4T1 cells was isolated using Trizol reagent (Invitrogen), following the manufacturer's recommendations. Using the TrueLabeling-AMP™ 2.0 kit, (Superarray Bioscience Corp., Frederick, Md.), the RNA was reversely transcribed to obtain cDNA and converted into biotin-labeled cRNA using biotin-16-UTP (Roche, Mannheim, Germany) by *in vitro *transcription. cRNA probes were then purified with the ArrayGrade cRNA cleanup kit (Superarray) and hybridized to the pretreated Oligo GEArray^® ^Mouse Tumor metastasis microarray (Superarray). Following washing steps, array spots binding cRNA were detected using alkaline phosphatase-conjugated streptavidin and CDP-Star as chemiluminescent substrate. Signal was detected by exposure to high-performance chemiluminescence films (Amersham Biosciences, UK). The image data were transformed into numerical data using GEArray Expression Analysis Suite software (SuperArray Bioscience). To normalize the data, background signal was subtracted and the intensity of all genes was referred to GAPDH as an endogenous control. Data filtering criteria were as follows: at least one of the spot intensities to be compared had to be more than twice the background intensity, and the spot intensity ratios had to be ± 40% in all set of samples analyzed to consider up or down-regulation. Finally, mean expression of each gene was transformed into color intensity using the program TIGRMultiExperiment Viewer V4.5.1.

### Western blot analysis

Western blot analysis for the detection of SMAD2 (sc-6200, Santa Cruz, US) and β-catenin (sc-7199, Santa Cruz) were performed. After treatment, cells were harvested and lysed in buffer, containing 1.5 mM Tris (Bio-Rad Labs), 150 mM NaCL, 0.1% SDS, 1% NP-40, 0.02% Sodium Azide, pH 8, with proteinase inhibitors 4% complete and 1% PMSF (phenylmethylsulfonyl fluoride) (all from Sigma) as previously described [55]. Cell lysates were sonicated for 4 seconds and solid cellular debris were removed by centrifugation at 12.000 rpm for 10 min. Lystates were stored at -80°C until use. 20 ug of lysate was loaded in a 12% SDS-polyacrylamide gel, transferred to nitrocellulose membranes and processed according to standard Western blotting procedures. To normalize for protein content the blots were stripped in buffer containing 62.5 mM Tris-HCl, pH 6.7, 2% SDS, 100 mM β-mercaptoethanol and stained with anti-tubulin antibody (T4026, Sigma). The concentration of each target protein was normalized *vs *tubulin. NIH image software (ImageJ) was used to quantify the intensity of each band.

### Immunofluorescence

4T1 cells were cultured at a concentration of 30.000 cells per well in 8-well chamber slides. After 24 h fresh medium supplemented with CRF at a concentration of 2 × 10^-8^M was added. After 2 or 4 h cells were fixed with 3.7% formaldehyde in PBS for 10 min, permeabilized with acetone for 4 min, washed with PBS and blocked with 1.5% FCS in PBS for 15 min. The chamber slides were subsequently incubated with rhodamine-phalloidin (Sigma) at a 1:100 dilution in 1.5% FCS in PBS, for 30 min at dark. Cells probed with rhodamine-phalloidin were washed with PBS and immediately mounted and stored at -20°C until observation with confocal laser scanning microscopy.

### Wound healing assay

Cells were cultured in 60 mm plates until they fromed a monolayer. A small area was then disrupted and a group of cells was destroyed or displaced by scratching a line through the layer with a tip [18]. The culture medium was replaced with serum free medium and cells received the stimulus (CRF and/or Antalarmin). The open gap was then inspected microscopically (Leica, Germany) over time as the cells moved in and filled the damaged area. Images were captured at the beginning and at regular time points during cell migration and the cell migration was quantified by measuring the distance with the program Image J http://rsbweb.nih.gov/ij/ between two certain points on either side of the gap. For proper statistical evaluation, at least three measurements at different points were performed at each image.

### Transfections and luciferase assay

4T1 cells were transfected with a pGL3 plasmid containing a WNT-reporter sequence harbouring 3 TCF binding sites linked to the luciferase by lipofectamine™ 2000 (Invitrogen), according to the manufacturer's instructions. In parallel experiments, the same plasmid was used that carries a point mutation in the TCF binding site. In brief, one day before transfection, cells were plated at 1,5 × 10^5 ^cells/well in 500 μl medium in 24-well plates. Plasmid DNA and lipofectamine both diluted in Opti-MEM I reduced serum medium without serum (Invitrogen), were mixed at a 1:2 ratio (1 μg DNA: 2 μl lipofectamine/well) and incubated for 20 min at room temperature. After 20 min incubation, 100 μl of plasmid/liposome complex was added to each well, and cells were incubated for 24 hours at 37°C and 5% CO_2_. Medium was refreshed after 4 hours with normal culture medium containing 10^-8 ^M CRF, cells were lysed after 18 hours. Luciferase assay was performed with the Dual-Luciferase^® ^Reporter Assay System (Promega), according to the manufacturer's manual. Each transfection was performed in triplicate to allow statistical evaluation and control for possible variations in transfection efficiency.

### RIA

Corticosterone was measured by RIA in serum collected at the indicated time points. Five animals per group were used. Sera were frozen at -70°C and analyzed as recommended by the manufacturer (ICN, USA).

### Animals

Six to eight weeks old Balb/c female mice were purchased from the Hellenic Pasteur Institute (Athens, Greece). All procedures described below were approved by the Animal Care Committee of the University of Crete School of Medicine, Heraklio, Crete, Greece and from the Veterinary Department of the Heraklion Prefecture, Heraklio, Crete, Greece.

### In vivo model of breast tumor and restraint stress

One million 4T1 cells were implanted in the mammary fat pad of Balb/c mice and three different groups were created. One group was injected intraperitoneally with 20 mg/kg antalarmin everyday. A second group was exposed to restraint stress for 3 hours for 4 consecutive days following a 5 day interval. The third group was exposed to the same type of restraint stress and in addition received 20 mg/kg antalarmin daily, dissolved in cremaphor (Sigma). Control group was subjected to the same surgical procedure, without the implantation of tumor cells.

We used antalarmin for inhibition of CRF receptors since CRF functions primarily via CRF_1_, the target of antalarmin. Mice that did not receive antalarmin received an injection of vehicle at the same time points. At different time points (either before or immediately after the restraint period) samples were collected by the retroorbital route to measure corticosterone in the plasma. The experiment was terminated 6 weeks later. At the end of the experiment mammary glands were visualized on the animal to determine the extent of neoangiogenesis and samples were collected from the different groups and histological analysis was performed.

### Angiogenesis determination

To quantitatively measure angiogenesis an in-house developed method was used. Briefly, this method utilized the contrast of autofluorescence between the mammary tissue and the blood vessels. Blood vessels greatly absorb visible light, while mammary gland and mammary tumors are strongly autofluorescent. User-friendly software developed in-house was used in order to quantify the area of vascularization *vs *total area.

### Histological analysis

Mammary glands samples were collected as specified above and fixed in formalin. Sections were stained with Haematoxylin-Eosin using standard techniques. Presence of tumors was determined by the same pathologist blinded to the treatment conditions. Percentage of tumor bearing mice was calculated per each group.

### Statistical analysis

Comparison between groups was made using the Student's *t*-test and ANOVA test, and *p *< 0.05 was considered significant.

## List of Abbreviations

CRF: Corticotropin Releasing Factor; CRF1, CRF2: Corticotropin Releasing Factor receptor 1, or receptor 2; UCN: Urocortin; WNT: wingless-type MMTV integration site family member 2; IL-: Interleukin; TNF: Tumor necrosis factor; TGF: Transforming growth factor; SMAD2: SMAD family member 2.

## Competing interests

The authors declare that they have no competing interests.

## Authors' contributions

AAr performed the in vivo studies, the gene expression array analysis, SMAD and β-catenin expression studies, the angiogenesis studies and drafted the manuscript. MV participated in the in vivo experiments, performed corticosterone measurements, participated in data evaluation, co-ordination of the study and manuscript preparation. BM performed SMAD experession analysis, cell proliferation studies and confocal studies. ED participated in confocal microscopy analyses and co-ordination of the study. AAn participated in the in vivo studies data evaluation and co-ordination of the study. OR participated in the cell proliferation studies and receptor expression analysis. ENS participated in the histological analysis and evaluation. RG participated in the array analysis and drafting the manuscript. JR designed the software and participated in the angiogenesis analysis. ANM participated in the design of the study and drafting of the manuscript. CT conceived of the study, participated in its design and drafted the manuscript. All authors read and approved the final manuscript.

## References

[B1] Hasegawa H Saiki I Psychosocial stress augments tumor development through beta-adrenergic activation in mice Jpn J Cancer Res 2002 93 729 735 12149137 10.1111/j.1349-7006.2002.tb01313.x PMC5927068

[B2] Reiche EM Nunes SO Morimoto HK Stress, depression, the immune system, and cancer Lancet Oncol 2004 5 617 625 10.1016/S1470-2045(04)01597-9 15465465

[B3] Tsatsanis C Dermitzaki E Venihaki M Chatzaki E Minas V Gravanis A Margioris AN The corticotropin-releasing factor (CRF) family of peptides as local modulators of adrenal function Cell Mol Life Sci 2007 64 1638 1655 10.1007/s00018-007-6555-7 17453142 PMC11136361

[B4] Kiank C Tache Y Larauche M Stress-related modulation of inflammation in experimental models of bowel disease and post-infectious irritable bowel syndrome: role of corticotropin-releasing factor receptors Brain Behav Immun 24 41 48 10.1016/j.bbi.2009.08.006 19698778 PMC2962412

[B5] Tache Y Perdue MH Role of peripheral CRF signalling pathways in stress-related alterations of gut motility and mucosal function Neurogastroenterol Motil 2004 16 Suppl 1 137 142 10.1111/j.1743-3150.2004.00490.x 15066020

[B6] Anton PM Gay J Mykoniatis A Pan A O'Brien M Brown D Karalis K Pothoulakis C Corticotropin-releasing hormone (CRH) requirement in Clostridium difficile toxin A-mediated intestinal inflammation Proc Natl Acad Sci USA 2004 101 8503 8508 10.1073/pnas.0402693101 15159534 PMC420423

[B7] Bale TL Hoshijima M Gu Y Dalton N Anderson KR Lee KF Rivier J Chien KR Vale WW Peterson KL The cardiovascular physiologic actions of urocortin II: acute effects in murine heart failure Proc Natl Acad Sci USA 2004 101 3697 3702 10.1073/pnas.0307324101 14990799 PMC373525

[B8] Nazarloo HP Buttrick PM Saadat H Dunn AJ The roles of corticotropin-releasing factor-related peptides and their receptors in the cardiovascular system Curr Protein Pept Sci 2006 7 229 239 10.2174/138920306777452358 16787262

[B9] Baigent SM Peripheral corticotropin-releasing hormone and urocortin in the control of the immune response Peptides 2001 22 809 820 10.1016/S0196-9781(01)00395-3 11337095

[B10] Arbiser JL Karalis K Viswanathan A Koike C Anand-Apte B Flynn E Zetter B Majzoub JA Corticotropin-releasing hormone stimulates angiogenesis and epithelial tumor growth in the skin J Invest Dermatol 1999 113 838 842 10.1046/j.1523-1747.1999.00760.x 10571742

[B11] Bale TL Vale WW CRF and CRF receptors: role in stress responsivity and other behaviors Annu Rev Pharmacol Toxicol 2004 44 525 557 10.1146/annurev.pharmtox.44.101802.121410 14744257

[B12] Kaprara A Pazaitou-Panayiotou K Kortsaris A Chatzaki E The corticotropin releasing factor system in cancer: expression and pathophysiological implications Cell Mol Life Sci 2010 67 1293 306 10.1007/s00018-010-0265-2 20143250 PMC11115652

[B13] Tezval H Jurk S Atschekzei F Becker JU Jahn O Serth J Kuczyk MA Urocortin and corticotropin-releasing factor receptor 2 in human renal cell carcinoma: disruption of an endogenous inhibitor of angiogenesis and proliferation World J Urol 2009 27 825 830 10.1007/s00345-009-0417-x PMC2780655 19437022

[B14] Willenberg HS Haase M Papewalis C Schott M Scherbaum WA Bornstein SR Corticotropin-releasing hormone receptor expression on normal and tumorous human adrenocortical cells Neuroendocrinology 2005 82 274 281 10.1159/000093126 16721033

[B15] Graziani G Ferrandina G Pozzoli G Vergati M Muzi A Legge F Tentori L Scambia G Navarra P Corticotropin-releasing hormone receptor-1 in human endometrial cancer Oncol Rep 2006 15 375 379 16391857

[B16] Tezval H Jurk S Atschekzei F Serth J Kuczyk MA Merseburger AS The involvement of altered corticotropin releasing factor receptor 2 expression in prostate cancer due to alteration of anti-angiogenic signaling pathways Prostate 2009 69 443 448 10.1002/pros.20892 19058138

[B17] Graziani G Tentori L Muzi A Vergati M Tringali G Pozzoli G Navarra P Evidence that corticotropin-releasing hormone inhibits cell growth of human breast cancer cells via the activation of CRH-R1 receptor subtype Mol Cell Endocrinol 2007 264 44 49 10.1016/j.mce.2006.10.006 17097220

[B18] Androulidaki A Dermitzaki E Venihaki M Karagianni E Rassouli O Andreakou E Stournaras C Margioris AN Tsatsanis C Corticotropin Releasing Factor promotes breast cancer cell motility and invasiveness Mol Cancer 2009 8 30 10.1186/1476-4598-8-30 19490624 PMC2697132

[B19] Minas V Rolaki A Kalantaridou SN Sidiropoulos J Mitrou S Petsas G Jeschke U Paraskevaidis EA Fountzilas G Chrousos GP Intratumoral CRH modulates immuno-escape of ovarian cancer cells through FasL regulation Br J Cancer 2007 97 637 645 10.1038/sj.bjc.6603918 17667919 PMC2360374

[B20] Venihaki M Gravanis A Margioris AN Comparative study between normal rat chromaffin and PC12 rat pheochromocytoma cells: production and effects of corticotropin-releasing hormone Endocrinology 1997 138 698 704 10.1210/en.138.2.698 9003004

[B21] Venihaki M Ain K Dermitzaki E Gravanis A Margioris AN KAT45, a noradrenergic human pheochromocytoma cell line producing corticotropin-releasing hormone Endocrinology 1998 139 713 722 10.1210/en.139.2.713 9449645

[B22] Sato H Nagashima Y Chrousos GP Ichihashi M Funasak Y The expression of corticotropin-releasing hormone in melanoma Pigment Cell Res 2002 15 98 103 10.1034/j.1600-0749.2002.1o063.x 11936276

[B23] Yang Y Park H Kim TS Bang SI Cho D Enhancement of cell migration by corticotropin-releasing hormone through ERK1/2 pathway in murine melanoma cell line, B16F10 Exp Dermatol 2007 16 22 27 10.1111/j.1600-0625.2006.00511.x 17181633

[B24] Graziani G Tentori L Portarena I Barbarino M Tringali G Pozzoli G Navarra P CRH inhibits cell growth of human endometrial adenocarcinoma cells via CRH-receptor 1-mediated activation of cAMP-PKA pathway Endocrinology 2002 143 807 813 10.1210/en.143.3.807 11861501

[B25] Slominski AT Roloff B Zbytek B Wei ET Fechner K Curry J Wortsman J Corticotropin releasing hormone and related peptides can act as bioregulatory factors in human keratinocytes In Vitro Cell Dev Biol Anim 2000 36 211 216 10.1290/1071-2690(2000)036<0211:CRHARP>2.0.CO;2 10777063

[B26] Radulovic M Hippel C Spiess J Corticotropin-releasing factor (CRF) rapidly suppresses apoptosis by acting upstream of the activation of caspases J Neurochem 2003 84 1074 1085 10.1046/j.1471-4159.2003.01594.x 12603831

[B27] Wong ML Webster EL Spokes H Phu P Ehrhart-Bornstein M Bornstein S Park CS Rice KC Chrousos GP Licinio J Gold PW Chronic administration of the non-peptide CRH type 1 receptor antagonist antalarmin does not blunt hypothalamic-pituitary-adrenal axis responses to acute immobilization stress Life Sci 1999 65 PL53 58 10.1016/S0024-3205(99)00268-4 10421433

[B28] Yingling JM Blanchard KL Sawyer JS Development of TGF-beta signalling inhibitors for cancer therapy Nat Rev Drug Discov 2004 3 1011 1022 10.1038/nrd1580 15573100

[B29] Gebeshuber CA Sladecek S Grunert S Beta-catenin/LEF-1 signalling in breast cancer--central players activated by a plethora of inputs Cells Tissues Organs 2007 185 51 60 10.1159/000101303 17587808

[B30] Carlier MF Pantaloni D Control of actin assembly dynamics in cell motility J Biol Chem 2007 282 23005 23009 10.1074/jbc.R700020200 17576764

[B31] Tsatsanis C Androulidaki A Dermitzaki E Gravanis A Margioris AN Corticotropin releasing factor receptor 1 (CRF1) and CRF2 agonists exert an anti-inflammatory effect during the early phase of inflammation suppressing LPS-induced TNF-alpha release from macrophages via induction of COX-2 and PGE2 J Cell Physiol 2007 210 774 783 10.1002/jcp.20900 17117478

[B32] Tsatsanis C Androulidaki A Venihaki M Margioris AN Signalling networks regulating cyclooxygenase-2 Int J Biochem Cell Biol 2006 38 1654 1661 10.1016/j.biocel.2006.03.021 16713323

[B33] Webster EL Lewis DB Torpy DJ Zachman EK Rice KC Chrousos GP In vivo and in vitro characterization of antalarmin, a nonpeptide corticotropin-releasing hormone (CRH) receptor antagonist: suppression of pituitary ACTH release and peripheral inflammation Endocrinology 1996 137 5747 5750 10.1210/en.137.12.5747 8940412

[B34] Agelaki S Tsatsanis C Gravanis A Margioris AN Corticotropin-releasing hormone augments proinflammatory cytokine production from macrophages in vitro and in lipopolysaccharide-induced endotoxin shock in mice Infect Immun 2002 70 6068 6074 10.1128/IAI.70.11.6068-6074.2002 12379683 PMC130344

[B35] Im E Rhee SH Park YS Fiocchi C Tache Y Pothoulakis C The Corticotropin Releasing Hormone Family of Peptides Regulates Intestinal Angiogenesis Gastroenterology 2010 138 2457 67 10.1053/j.gastro.2010.02.055 20206175 PMC2883634

[B36] Saez Mdel C Barriga C Garcia JJ Rodriguez AB Ortega E Exercise-induced stress enhances mammary tumor growth in rats: beneficial effect of the hormone melatonin Mol Cell Biochem 2007 294 19 24 10.1007/s11010-005-9067-5 17136443

[B37] Kalantaridou S Makrigiannakis A Zoumakis E Chrousos GP Peripheral corticotropin-releasing hormone is produced in the immune and reproductive systems: actions, potential roles and clinical implications Front Biosci 2007 12 572 580 10.2741/2083 17127318

[B38] Paus R Theoharides TC Arck PC Neuroimmunoendocrine circuitry of the 'brain-skin connection' Trends Immunol 2006 27 32 39 10.1016/j.it.2005.10.002 16269267

[B39] Parkes DG Weisinger RS May CN Cardiovascular actions of CRH and urocortin: an update Peptides 2001 22 821 827 10.1016/S0196-9781(01)00396-5 11337096

[B40] van Wijk PA van Neck JW Rijnberk A Croughs RJ Mol JA Proliferation of the murine corticotropic tumour cell line AtT20 is affected by hypophysiotrophic hormones, growth factors and glucocorticoids Mol Cell Endocrinol 1995 111 13 19 10.1016/0303-7207(95)03541-E 7544306

[B41] van Wijk PA Rijnberk A Croughs RJ Meij BP Mol JA Effects of corticotrophin-releasing hormone, vasopressin and insulin-like growth factor-I on proliferation of and adrenocorticotrophic hormone secretion by canine corticotrophic adenoma cells in vitro Eur J Endocrinol 1998 138 309 315 10.1530/eje.0.1380309 9539306

[B42] Slominski A Zbytek B Pisarchik A Slominski RM Zmijewski MA Wortsman J CRH functions as a growth factor/cytokine in the skin J Cell Physiol 2006 206 780 791 10.1002/jcp.20530 16245303 PMC1351367

[B43] Gavert N Ben-Ze'ev A beta-Catenin signaling in biological control and cancer J Cell Biochem 2007 102 820 828 10.1002/jcb.21505 17854061

[B44] Hirota M Watanabe K Hamada S Sun Y Strizzi L Mancino M Nagaoka T Gonzales M Seno M Bianco C Salomon DS Smad2 functions as a co-activator of canonical Wnt/beta-catenin signaling pathway independent of Smad4 through histone acetyltransferase activity of p300 Cell Signal 2008 20 1632 1641 10.1016/j.cellsig.2008.05.003 18595660 PMC2578836

[B45] Olson MF Sahai E The actin cytoskeleton in cancer cell motility Clin Exp Metastasis 2009 26 273 287 10.1007/s10585-008-9174-2 18498004

[B46] Cassoni P Sapino A Negro F Bussolati G Oxytocin inhibits proliferation of human breast cancer cell lines Virchows Arch 1994 425 467 472 10.1007/BF00197549 7850070

[B47] Cassoni P Papotti M Ghe C Catapano F Sapino A Graziani A Deghenghi R Reissmann T Ghigo E Muccioli G Identification, characterization, and biological activity of specific receptors for natural (ghrelin) and synthetic growth hormone secretagogues and analogs in human breast carcinomas and cell lines J Clin Endocrinol Metab 2001 86 1738 1745 10.1210/jc.86.4.1738 11297611

[B48] Jeffery PL Murray RE Yeh AH McNamara JF Duncan RP Francis GD Herington AC Chopin LK Expression and function of the ghrelin axis, including a novel preproghrelin isoform, in human breast cancer tissues and cell lines Endocr Relat Cancer 2005 12 839 850 10.1677/erc.1.00984 16322325

[B49] Wlk M Wang CC Venihaki M Liu J Zhao D Anton PM Mykoniatis A Pan A Zacks J Karalis K Pothoulakis C Corticotropin-releasing hormone antagonists possess anti-inflammatory effects in the mouse ileum Gastroenterology 2002 123 505 515 10.1053/gast.2002.34783 12145804

[B50] Webster EL Barrientos RM Contoreggi C Isaac MG Ligier S Gabry KE Chrousos GP McCarthy EF Rice KC Gold PW Sternberg EM Corticotropin releasing hormone (CRH) antagonist attenuates adjuvant induced arthritis: role of CRH in peripheral inflammation J Rheumatol 2002 29 1252 1261 12064844

[B51] Hao Z Huang Y Cleman J Jovin IS Vale WW Bale TL Giordano FJ Urocortin2 inhibits tumor growth via effects on vascularization and cell proliferation Proc Natl Acad Sci USA 2008 105 3939 3944 10.1073/pnas.0712366105 18308934 PMC2268793

[B52] Wang J Xu Y Zhu H Zhang R Zhang G Li S Urocortin's inhibition of tumor growth and angiogenesis in hepatocellular carcinoma via corticotrophin-releasing factor receptor 2 Cancer Invest 2008 26 359 368 10.1080/07357900701788106 18443956

[B53] Theoharides TC Singh LK Boucher W Pang X Letourneau R Webster E Chrousos G Corticotropin-releasing hormone induces skin mast cell degranulation and increased vascular permeability, a possible explanation for its proinflammatory effects Endocrinology 1998 139 403 413 10.1210/en.139.1.403 9421440

[B54] Arranz A Androulidaki A Zacharioudaki V Martinez C Margioris AN Gomariz RP Tsatsanis C Vasoactive intestinal peptide suppresses toll-like receptor 4 expression in macrophages via Akt1 reducing their responsiveness to lipopolysaccharide Mol Immunol 2008 45 2970 2980 10.1016/j.molimm.2008.01.023 18336909

[B55] Androulidaki A Iliopoulos D Arranz A Doxaki C Schworer S Zacharioudaki V Margioris AN Tsichlis PN Tsatsanis C The kinase Akt1 controls macrophage response to lipopolysaccharide by regulating microRNAs Immunity 2009 31 220 231 10.1016/j.immuni.2009.06.024 19699171 PMC2865583

